# Supplementary material for: Modeling glioblastoma heterogeneity as a dynamic network of cell states
Source: Mol Syst Biol. 2021 Sep 16;17(9):e10105. doi: 10.15252/msb.202010105 (PMC8444284; doi:10.15252/msb.202010105)
Supplement: Supplementary file 5 — Source Data for Figure 3 [file MSB-17-e10105-s001.zip › Figure3A_sourcedata/GSEA_3065/hallmarks_state6.GseaPreranked.1623416308702/HALLMARK_DNA_REPAIR.html]

Details for gene set HALLMARK\_DNA\_REPAIR[GSEA]

|  || Dataset | state6 |
| Phenotype | NoPhenotypeAvailable |
| Upregulated in class | na\_pos |
| GeneSet | HALLMARK\_DNA\_REPAIR |
| Enrichment Score (ES) | 0.25676823 |
| Normalized Enrichment Score (NES) | 1.1470927 |
| Nominal p-value | 0.17894737 |
| FDR q-value | 0.32402417 |
| FWER p-Value | 0.987 |
Table: GSEA Results Summary

  

Fig 1: Enrichment plot: HALLMARK\_DNA\_REPAIR      
 Profile of the Running ES Score & Positions of GeneSet Members on the Rank Ordered List

  

| PROBE | GENE SYMBOL | GENE\_TITLE | RANK IN GENE LIST | RANK METRIC SCORE | RUNNING ES | CORE ENRICHMENT || 1 | GUK1 |  |  | 93 | 0.218 | 0.0175 | Yes |
| 2 | EIF1B |  |  | 112 | 0.207 | 0.0422 | Yes |
| 3 | TP53 |  |  | 116 | 0.205 | 0.0684 | Yes |
| 4 | POLR2J |  |  | 190 | 0.180 | 0.0832 | Yes |
| 5 | NME4 |  |  | 309 | 0.149 | 0.0889 | Yes |
| 6 | POLR2F |  |  | 348 | 0.144 | 0.1032 | Yes |
| 7 | COX17 |  |  | 361 | 0.142 | 0.1201 | Yes |
| 8 | GPX4 |  |  | 365 | 0.141 | 0.1380 | Yes |
| 9 | TAF13 |  |  | 372 | 0.140 | 0.1554 | Yes |
| 10 | RALA |  |  | 374 | 0.140 | 0.1734 | Yes |
| 11 | RFC4 |  |  | 397 | 0.136 | 0.1885 | Yes |
| 12 | TAF12 |  |  | 399 | 0.136 | 0.2060 | Yes |
| 13 | TAF10 |  |  | 649 | 0.110 | 0.1915 | Yes |
| 14 | POLB |  |  | 699 | 0.106 | 0.1995 | Yes |
| 15 | RRM2B |  |  | 761 | 0.101 | 0.2056 | Yes |
| 16 | SAC3D1 |  |  | 860 | 0.094 | 0.2064 | Yes |
| 17 | DGUOK |  |  | 894 | 0.091 | 0.2144 | Yes |
| 18 | VPS28 |  |  | 1013 | 0.085 | 0.2118 | Yes |
| 19 | IMPDH2 |  |  | 1043 | 0.083 | 0.2192 | Yes |
| 20 | POLR3GL |  |  | 1045 | 0.083 | 0.2299 | Yes |
| 21 | SSRP1 |  |  | 1124 | 0.080 | 0.2312 | Yes |
| 22 | SNAPC5 |  |  | 1322 | 0.070 | 0.2176 | Yes |
| 23 | TMED2 |  |  | 1445 | 0.066 | 0.2121 | Yes |
| 24 | RBX1 |  |  | 1446 | 0.066 | 0.2206 | Yes |
| 25 | POLR2C |  |  | 1564 | 0.062 | 0.2151 | Yes |
| 26 | ITPA |  |  | 1572 | 0.061 | 0.2223 | Yes |
| 27 | APRT |  |  | 1573 | 0.061 | 0.2302 | Yes |
| 28 | TAF9 |  |  | 1644 | 0.059 | 0.2297 | Yes |
| 29 | POLR2I |  |  | 1716 | 0.056 | 0.2288 | Yes |
| 30 | MPG |  |  | 1718 | 0.056 | 0.2360 | Yes |
| 31 | POLR2K |  |  | 1720 | 0.056 | 0.2431 | Yes |
| 32 | GSDME |  |  | 1780 | 0.054 | 0.2433 | Yes |
| 33 | GTF3C5 |  |  | 1824 | 0.053 | 0.2452 | Yes |
| 34 | GTF2A2 |  |  | 1864 | 0.052 | 0.2474 | Yes |
| 35 | SUPT4H1 |  |  | 1869 | 0.052 | 0.2536 | Yes |
| 36 | AAAS |  |  | 2000 | 0.048 | 0.2448 | Yes |
| 37 | PDE4B |  |  | 2004 | 0.048 | 0.2506 | Yes |
| 38 | DDB2 |  |  | 2045 | 0.046 | 0.2520 | Yes |
| 39 | NFX1 |  |  | 2134 | 0.044 | 0.2475 | Yes |
| 40 | GTF2H1 |  |  | 2146 | 0.043 | 0.2518 | Yes |
| 41 | SURF1 |  |  | 2163 | 0.043 | 0.2555 | Yes |
| 42 | POLR2E |  |  | 2200 | 0.042 | 0.2568 | Yes |
| 43 | SUPT5H |  |  | 2303 | 0.038 | 0.2500 | No |
| 44 | TARBP2 |  |  | 2367 | 0.037 | 0.2475 | No |
| 45 | GTF2H3 |  |  | 2510 | 0.033 | 0.2354 | No |
| 46 | MPC2 |  |  | 2526 | 0.033 | 0.2379 | No |
| 47 | RPA3 |  |  | 2563 | 0.032 | 0.2379 | No |
| 48 | TSG101 |  |  | 2565 | 0.032 | 0.2418 | No |
| 49 | NELFB |  |  | 2799 | 0.027 | 0.2184 | No |
| 50 | SEC61A1 |  |  | 2806 | 0.026 | 0.2212 | No |
| 51 | POLR1D |  |  | 2819 | 0.026 | 0.2232 | No |
| 52 | EDF1 |  |  | 2913 | 0.024 | 0.2155 | No |
| 53 | SDCBP |  |  | 2964 | 0.023 | 0.2127 | No |
| 54 | POLR2A |  |  | 2984 | 0.022 | 0.2134 | No |
| 55 | POLR2D |  |  | 3013 | 0.022 | 0.2130 | No |
| 56 | POM121 |  |  | 3071 | 0.021 | 0.2090 | No |
| 57 | SRSF6 |  |  | 3114 | 0.020 | 0.2067 | No |
| 58 | ERCC3 |  |  | 3166 | 0.018 | 0.2032 | No |
| 59 | CETN2 |  |  | 3175 | 0.018 | 0.2047 | No |
| 60 | POLR1C |  |  | 3320 | 0.015 | 0.1901 | No |
| 61 | POLR2G |  |  | 3346 | 0.015 | 0.1891 | No |
| 62 | USP11 |  |  | 3393 | 0.013 | 0.1855 | No |
| 63 | POLA1 |  |  | 3456 | 0.012 | 0.1798 | No |
| 64 | TAF1C |  |  | 3482 | 0.012 | 0.1784 | No |
| 65 | PRIM1 |  |  | 3532 | 0.011 | 0.1742 | No |
| 66 | DUT |  |  | 3596 | 0.009 | 0.1681 | No |
| 67 | RFC2 |  |  | 3618 | 0.009 | 0.1669 | No |
| 68 | ALYREF |  |  | 3755 | 0.006 | 0.1519 | No |
| 69 | ERCC1 |  |  | 3807 | 0.005 | 0.1466 | No |
| 70 | TK2 |  |  | 3876 | 0.003 | 0.1392 | No |
| 71 | ERCC2 |  |  | 3957 | 0.001 | 0.1302 | No |
| 72 | DDB1 |  |  | 4082 | -0.001 | 0.1160 | No |
| 73 | POLE4 |  |  | 4140 | -0.002 | 0.1096 | No |
| 74 | ERCC4 |  |  | 4248 | -0.004 | 0.0978 | No |
| 75 | VPS37B |  |  | 4441 | -0.008 | 0.0767 | No |
| 76 | RAE1 |  |  | 4482 | -0.008 | 0.0732 | No |
| 77 | RFC5 |  |  | 4594 | -0.011 | 0.0617 | No |
| 78 | MRPL40 |  |  | 4631 | -0.011 | 0.0590 | No |
| 79 | UPF3B |  |  | 4763 | -0.014 | 0.0457 | No |
| 80 | SMAD5 |  |  | 4873 | -0.016 | 0.0352 | No |
| 81 | CANT1 |  |  | 4955 | -0.017 | 0.0281 | No |
| 82 | POLR2H |  |  | 5073 | -0.020 | 0.0172 | No |
| 83 | REV3L |  |  | 5120 | -0.021 | 0.0146 | No |
| 84 | ELOA |  |  | 5124 | -0.021 | 0.0169 | No |
| 85 | SNAPC4 |  |  | 5160 | -0.021 | 0.0157 | No |
| 86 | POLA2 |  |  | 5164 | -0.022 | 0.0181 | No |
| 87 | NELFCD |  |  | 5271 | -0.024 | 0.0089 | No |
| 88 | NCBP2 |  |  | 5373 | -0.026 | 0.0006 | No |
| 89 | SF3A3 |  |  | 5467 | -0.028 | -0.0065 | No |
| 90 | NME1 |  |  | 5479 | -0.028 | -0.0041 | No |
| 91 | HPRT1 |  |  | 5723 | -0.033 | -0.0279 | No |
| 92 | XPC |  |  | 5756 | -0.034 | -0.0272 | No |
| 93 | AK3 |  |  | 5846 | -0.035 | -0.0329 | No |
| 94 | DCTN4 |  |  | 5895 | -0.036 | -0.0338 | No |
| 95 | UMPS |  |  | 6019 | -0.039 | -0.0430 | No |
| 96 | RFC3 |  |  | 6078 | -0.040 | -0.0446 | No |
| 97 | GTF2B |  |  | 6424 | -0.048 | -0.0781 | No |
| 98 | NT5C |  |  | 6480 | -0.049 | -0.0781 | No |
| 99 | RPA2 |  |  | 6501 | -0.050 | -0.0740 | No |
| 100 | BCAM |  |  | 6610 | -0.053 | -0.0796 | No |
| 101 | GTF2H5 |  |  | 6633 | -0.054 | -0.0752 | No |
| 102 | CSTF3 |  |  | 6656 | -0.054 | -0.0707 | No |
| 103 | NME3 |  |  | 6728 | -0.057 | -0.0715 | No |
| 104 | ADCY6 |  |  | 7053 | -0.066 | -0.1003 | No |
| 105 | FEN1 |  |  | 7218 | -0.071 | -0.1100 | No |
| 106 | GTF2F1 |  |  | 7283 | -0.074 | -0.1078 | No |
| 107 | ZWINT |  |  | 7370 | -0.077 | -0.1078 | No |
| 108 | TAF6 |  |  | 7401 | -0.078 | -0.1012 | No |
| 109 | NUDT21 |  |  | 7524 | -0.082 | -0.1046 | No |
| 110 | GMPR2 |  |  | 7758 | -0.093 | -0.1194 | No |
| 111 | NT5C3A |  |  | 7839 | -0.098 | -0.1159 | No |
| 112 | ADRM1 |  |  | 7955 | -0.104 | -0.1157 | No |
| 113 | LIG1 |  |  | 7987 | -0.106 | -0.1055 | No |
| 114 | NELFE |  |  | 8020 | -0.108 | -0.0952 | No |
| 115 | POLD3 |  |  | 8033 | -0.109 | -0.0824 | No |
| 116 | PCNA |  |  | 8061 | -0.111 | -0.0712 | No |
| 117 | RNMT |  |  | 8277 | -0.129 | -0.0793 | No |
| 118 | TYMS |  |  | 8287 | -0.131 | -0.0634 | No |
| 119 | NUDT9 |  |  | 8429 | -0.150 | -0.0603 | No |
| 120 | DAD1 |  |  | 8535 | -0.169 | -0.0505 | No |
| 121 | DGCR8 |  |  | 8536 | -0.169 | -0.0286 | No |
| 122 | ARL6IP1 |  |  | 8628 | -0.203 | -0.0129 | No |
| 123 | BCAP31 |  |  | 8710 | -0.248 | 0.0099 | No |
Table: GSEA details [plain text format]

  

Fig 2: HALLMARK\_DNA\_REPAIR: Random ES distribution      
 Gene set null distribution of ES for **HALLMARK\_DNA\_REPAIR**
